# Opposite Regulation of Homer Signal at the NMJ Postsynaptic Micro Domain between Slow- and Fast-Twitch Muscles in an Experimentally Induced Autoimmune Myasthenia Gravis (EAMG) Mouse Model

**DOI:** 10.3390/ijms232315052

**Published:** 2022-11-30

**Authors:** Martin Schubert, Andreas Pelz, Gabor Trautmann, Katharina Block, Sandra Furlan, Martina Gutsmann, Siegfried Kohler, Pompeo Volpe, Dieter Blottner, Andreas Meisel, Michele Salanova

**Affiliations:** 1 Institute of Integrative Neuroanatomy, Charité—Universitätsmedizin Berlin, Corporate Member of Freie Universität Berlin, Humboldt-Universität zu Berlin and Berlin Institute of Health, 10115 Berlin, Germany; 2 Department of Experimental Neurology, Charité—Universitätsmedizin Berlin, 10115 Berlin, Germany; 3 Center of Space Medicine Berlin, Neuromuscular Signaling and System, 10115 Berlin, Germany; 4 C.N.R. Institute of Neuroscience, 35131 Padova, Italy; 5 Department of Biomedical Sciences, University of Padova, 35122 Padova, Italy

**Keywords:** skeletal muscle, Homer isoform expression, NMJ adaptation, Myasthenia Gravis

## Abstract

Accelerated postsynaptic remodelling and disturbance of neuromuscular transmission are common features of autoimmune neurodegenerative diseases. Homer protein isoform expression, crosslinking activity and neuromuscular subcellular localisation are studied in mouse hind limb muscles of an experimentally induced autoimmune model of Myasthenia Gravis (EAMG) and correlated to motor end plate integrity. *Soleus* (*SOL*), *extensor digitorum longus* (*EDL*) and *gastrocnemius* (*GAS*) skeletal muscles are investigated. nAChR membrane clusters were studied to monitor neuromuscular junction (NMJ) integrity. Fibre-type cross-sectional area (CSA) analysis is carried out in order to determine the extent of muscle atrophy. Our findings clearly showed that crosslinking activity of Homer long forms (Homer 1b/c and Homer2a/b) are decreased in slow-twitch and increased in fast-twitch muscle of EAMG whereas the short form of Homer that disrupts Homer crosslinking (Homer1a) is upregulated in slow-twitch muscle only. Densitometry analysis showed a 125% increase in Homer protein expression in *EDL*, and a 45% decrease in *SOL* of EAMG mice. In contrast, nAChR fluorescence pixel intensity decreased in endplates of EAMG mice, more distinct in type-I dominant *SOL* muscle. Morphometric CSA of EAMG vs. control (CTR) revealed a significant reduction in *EDL* but not in *GAS* and *SOL*. Taken together, these results indicate that postsynaptic Homer signalling is impaired in slow-twitch *SOL* muscle from EAMG mice and provide compelling evidence suggesting a functional coupling between Homer and nAChR, underscoring the key role of Homer in skeletal muscle neurophysiology.

## 1. Introduction

Specific isoforms of the Homer protein family are localised at the postsynaptic density of both central [1] and peripheral synapses [2], where they play a key role in synaptic plasticity [3,4], coordinating protein–protein interaction and cross-talk of different molecular players within a given or multiple postsynaptic signal transduction pathways [5].

Altogether, three different Homer genes have been identified with at least 20 isoforms [5] which are ubiquitously expressed. Homer long isoforms, i.e., Homer 1b and 1c, Homer 2, Homer 3, form large networks via a carboxyl-terminal coiled-coil (CC) region [6] and are constitutively expressed. Homer 1a, the inducible Homer short isoform, lacks the coiled-coil region and thus acts as a dominant-negative isoform by disrupting dimerisation/multimerisation of long Homer isoforms, uncoupling the scaffolding complex, and thus interrupting the interaction between Homer and its partners [1]. Different isoform expression is the result of alternative splicing [7]. An upregulation of short Homer 1a is induced upon high synaptic activity (e.g., seizures and/or hyper excitability) [1].

Homer proteins are present in mouse, rat, and human skeletal muscles and regulated by nerve and muscle activity [2,8]. For instance, Homer gene transcription, protein expression and subcellular localisation at the NMJ postsynaptic microdomain were downregulated in all human and animal models of disuse-induced muscle atrophy [2,9,10]. Moreover, reciprocal expression of Homer 1a and Homer 2 isoform is proposed as a key mechanism responsible for soleus muscle atrophy during chronic exposure to microgravity in space-flown mice [9].

Therefore, if confirmed by further studies, the altered expression pattern of Homer isoforms and modified cross-linking capacity could represent a general key pathogenetic mechanism of skeletal muscle diseases likely sharing the common trait of a deranged neuromuscular transmission.

One of the most common disorders of neuromuscular transmission is Myasthenia Gravis (MG), a chronic and progressive autoimmune disorder caused by the presence of autoantibodies directed against components of the postsynaptic muscular membrane, leading to muscular weakness mediated by a decreased NMJ function.

In the present study, we used a well-established animal experimental model of autoimmune myasthenia gravis (EAMG). This model mimics the pathology of the disease by immunising mice with purified exogenous nicotinic acetylcholine receptor (nAChR)-antigen, thus promoting an autoimmune response against their own nAChR [11]. In this experimental model we investigated the isoform expression pattern of Homer protein in slow- and fast-twitch skeletal muscle and evaluated motor endplate integrity.

Human MG, first described around 1900 [12], manifests itself with symptoms of muscle weakness, especially after repetitive muscle contraction, fatigue, and atrophy. Most patients (and some exclusively) show an impairment of ocular muscle function, presenting symptoms like ptosis and diplopia [13]. The disease progresses over time and without treatment can ultimately lead to paralysis of the respiratory-active diaphragm and end lethally. MG has a prevalence of 150–250 cases per 1 million and an incidence of 8–10 cases per 1 million [14].

Although the MG pathogenetic mechanisms at the postsynaptic membrane level have been extensively investigated, very little it is known about the molecular mechanisms and changes in protein complexes further downstream, which form the postsynaptic density (PSD) and regulate postsynaptic signal transduction.

As part of the postsynaptic signal transduction machinery, Homer was proposed to play a role in synaptic plasticity [15] and transmission strength fine-tuning [16], as well as causing muscle strength impairment when not being properly expressed [17]. Homer modulates Ca^2+^ influx through direct binding to the intracellular calcium release channel RyR1 and increase of channel open probability (*P0*) [18].

We hypothesised that the altered expression of specific Homer isoforms and thus altered cross-linking capacity at the subcellular level in the postsynaptic microdomain of the NMJ underlie the molecular mechanisms responsible for the disruption of synaptic transmission in MG.

The goal of this study was to investigate a possible link between Homer expression, subcellular localisation and cross-linking capacity, and the impairment of neuromuscular synaptic transmission. This unveils the functional coupling of Homer isoforms to postsynaptic nAChR.

## 2. Results

### 2.1. Endplate Destruction Is More Prominent in SOL Muscle

**Figure 1:**
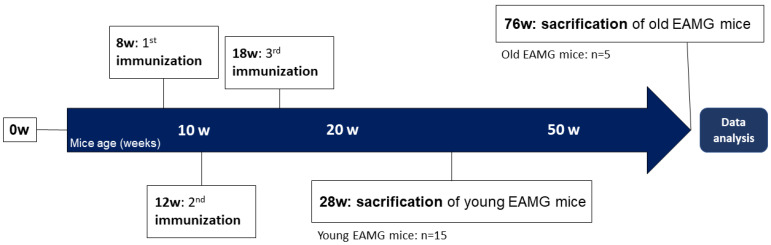
Diagram representing the EAMG study design. An age matched group of mice served as a reference control (CTR, *n* = 9) group without tAChR-antigen immunisation. W = weeks.

To monitor the effects of Myasthenia Gravis, signs of end plate destruction caused by circulating autoantibodies against junctional nAChRs are examined at histochemical and morphometric level in EAMG mice. Figure 1 shows a diagram representing the experimental study design and marks the time points at which mice are sacrificed for muscle dissection to study acute and chronic EAMG effects.

**Figure 2:**
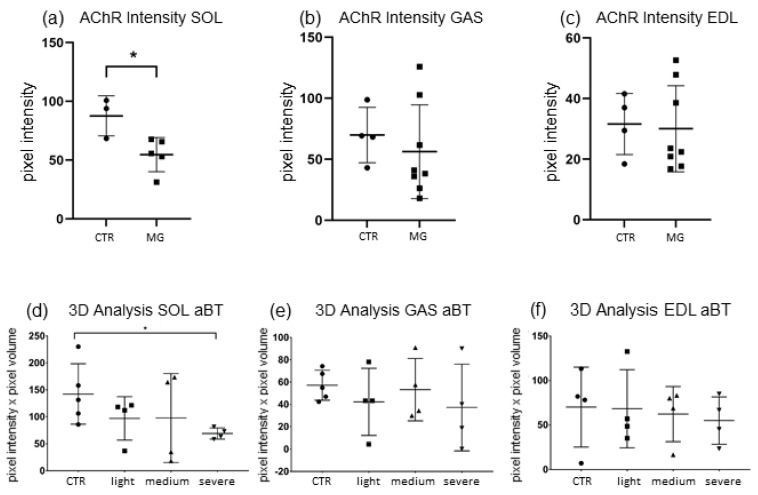
nAChR degradation rate in endplates of hindlimb muscles EAMG vs. CTR (**a**–**c**) and 3D-analysis of endplate nAChR-volume and pixel intensity, divided by clinical disease severity (**d**–**f**). Results of confocal pixel intensity analysis, Alpha Bungarotoxin (αBTX) staining. (**a**) Significant decrease in pixel intensity was present in *SOL* (−40.8%, *p* ≤ 0.033) of EAMG. (**b**) No significant changes were present in EDL and (**c**) *GAS*. (**d**) nAChR-amount was reduced significantly in SOL (48.2 %, *p* ≤ 0.0159) in severely affected mice. (**e**) No significant decrease was present in *GAS* and (**f**) in *EDL*. Data are express as means ± SD. For each experimental group, *n* = 4 male C57BL/N6 mice (28 weeks old). Statistical differences between groups were determined by Mann–Whitney-U-Test. * = indicates significant difference vs. CTR (ANOVA *p* ≤ 0.05). * indicates significant difference vs. CTR.

As shown in Figure 2a–c, a significant decrease of α-Bungarotoxin-stained nAChR pixel intensity was detected in *SOL* NMJs of EAMG-affected mice (−40.8%, *p* ≤ 0.033) when compared to CTR. Conversely, both *GAS* and *EDL* both displayed a lower mean end plate pixel intensity. However, no significant differences between EAMG (*GAS* −32.4%, *p* ≤ 0.28; *EDL* −30.08%, *p* ≤ 0.80) vs. CTR samples were present.

Overall, high individual differences of end plate affection between NMJ samples of MG mice are observed, ranging from almost no detectable staining at all to similar staining in EAMG and CTR. This suggests a variable degree of EAMG disease progression in each mice group depending on the different vulnerability of each individual mouse.

3D-analysis of NMJs was done on the second batch of EAMG mice, consisting of 12 young animals with different clinical disease severity, divided in three groups depending on clinical scoring (Group I = CTR, Group II = Score 0, Group III = Score 1, Group IV = Score 2) (For clinical scoring see Section 4).

The total volume as well as mean pixel intensity of NMJs was determined to quantify the amount of nAChRs accumulated in scanned endplates by multiplying those two parameters. A comparison between EAMG and CTR as well as in between clinical groups (I–IV) is carried out to ascertain whether there was a correlation between severity of symptoms and endplate destruction.

3D-analysis showed similar results as pixel intensity (Figure 2d–f). In *SOL*, nAChR quantity was reduced significantly only in severely Group IV EAMG affected mice compared to CTR (48.2%, *p* ≤ 0.0159). However, when comparing all EAMG-samples to CTR, no significant result could be shown (*p* ≤ 0.1037), even though mean nAChR-quantity was reduced more than in the other muscles (−41.3% CTR vs. EAMG). In *EDL* and *GAS* however, no significant reduction of nAChR-quantity could be detected, neither in between EAMG-affected animals itself nor in comparison to CTR mice muscles (*GAS* −24.14% CTR vs. EAMG *p* ≤ 0.33; *EDL* −20.96% CTR vs. EAMG, *p* ≤ 0.88).

In an attempt to address whether ageing differences were present, we compared young (approx. 30w) to old EAMG animal muscles (ca. 78w). Surprisingly, no significant changes between end plate affection in these groups could be detected, although in all three muscles chronically affected EAMG mice showed slight sign of nAChR-degradation progression (mean pixel intensity reduction found in old vs. young mice: *GAS* −39%, *EDL* −26%, *SOL* −16%).

### 2.2. Homer Protein Expression Is Affected Differently in SOL Than in GAS or EDL

Since EAMG muscles displayed a difference in endplate affection between slow-fibre dominant muscle *SOL* and the fast-fibre dominant muscles *GAS* and *EDL*, we then carried out Homer protein immunodetection at the NMJ and mRNA transcription in these two muscle groups. A 3D-analysis of Homer staining was carried out, again quantifying both volume and mean pixel intensity of endplate-adjacent Homer-staining. To reduce interference of unrelated background influence, only samples with a minimal difference of 10% mean pixel intensity in endplate staining compared to a randomly chosen background area were included for analysis.

**Figure 3:**
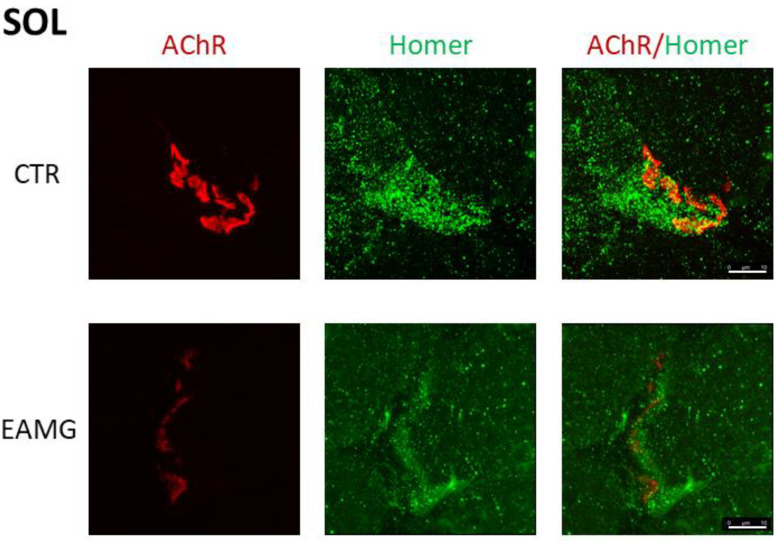
Representative immunohistochemical staining of *SOL* muscle endplates and endplate-adjacent Homer protein. *SOL* endplate-AChRs stained with α-Bungarotoxin (**red**), Homer protein stained with Pan-Homer-antibody (**green**). Right picture: merged AChR- and Homer-staining. Upper panel represents CTR-group, lower panel EAMG group. Confocal images display maximum projection of z-stacks of endplates scanned. Z-stacks are merged via 3D-analysis software and analysed for pixel intensity and volume. Magnification bar = 10 µm.

In Figure 3 we show representative confocal images of *SOL* muscle cryosections from both CTR and EAMG Group IV mice, double-stained with a panHomer or isoform unselective antibody and α-Bungarotoxin. A large positive postsynaptic microdomain is detectable in CTR cryosections not present in EAMG *SOL* muscle (Figure 3).

**Figure 4:**
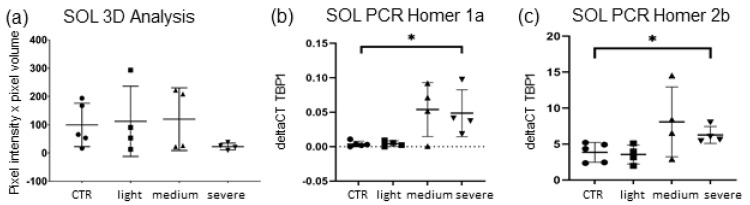
Homer protein isoform expression in *SOL* and *EDL* muscle. (**a**): 3D-analysis of endplate Homer pixel intensity and volume. A trend of reduction can be seen in severely affected EAMG mice in Homer quantity compared to CTR (−37%, *p* ≤ 0.111). (**b**,**c**): By qPCR-analysis, a significant increase of mRNA was detected in *SOL*-muscle for short isoform 1a (+932%, *p* ≤ 0.021) and long isoform Homer 2 (+62%, *p* ≤ 0.027). Homer 2b increase is lower than 1a-increase. Both times mRNA elevation was attested in severely affected mice, in medium affected mice only a trend was observed. Data are normalised to TATA-box-binding protein (TBP1) and expressed as means ± SD. Mann–Whitney-U test, significance level *p* ≤ 0.05. * indicates significant difference vs. CTR.

3D endplate quantification analysis was complemented by a qPCR gene analysis of each hindlimb muscle, quantifying the Homer gene expression regarding different isoforms of Homer (Figure 2a–c and Figure 4). The animals were again divided by clinical subgroups and compared to CTR.

As shown, *SOL* muscle 3D analysis of Homer showed no significant results between EAMG and CTR muscle, even though mean Homer quantity was lower (−51.35%) in EAMG animals and even less (−65.3%) when only considering severely (Group IV) affected mice (Figure 4a). This group also displayed the largest decrease in nAChR-quantity in 3D analysis as described above (Figure 2a), linking a more severe endplate destruction with a higher decrease of endplate-adjacent Homer protein in *SOL*.

qPCR gene expression analysis of *SOL* muscle showed a significant increase of expression in short isoform Homer 1a in severely affected mice (IV) with an increment of over 900% (*p* ≤ 0.021) (Figure 4b). The spike in mRNA of Homer 1a was significant in group IV as well as group III mice (medium clinical symptoms). Since basal expression of Homer 1a in *SOL* is very low, quantitation is characterised by a high dispersion and higher rates of uncertainty. In addition to Homer 2a, a significant increase of long isoform Homer 2 was detected, displaying a gain in mRNA (+62% in group IV, +95% in group III) compared to CTR, reaching significance only in group IV (*p* ≤ 0.027) (Figure 4c).

### 2.3. No Significant Change in Endplate-Adjacent Homer Quantity in Fast-Type Dominant Muscles

**Figure 5:**
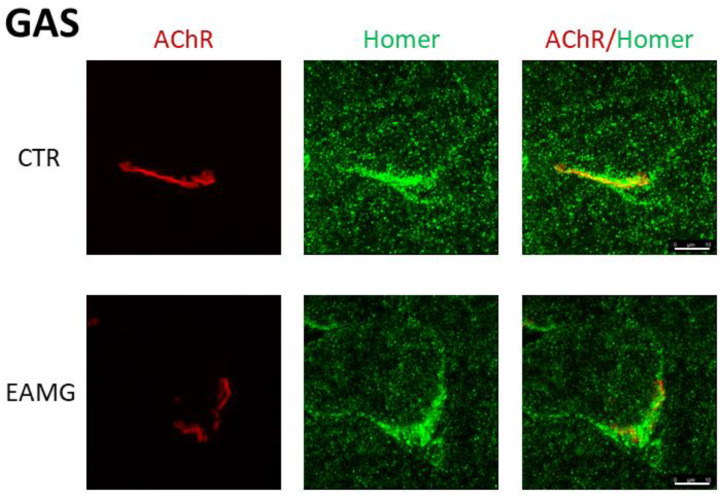

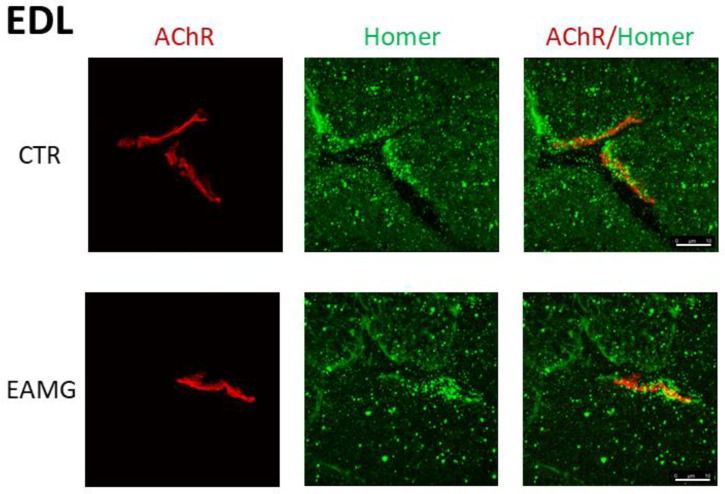
Representative immunohistochemical staining of *GAS* and *EDL* muscle endplates and endplate-adjacent Homer protein. For each image block (GAS, EDL) upper panel CTR, lower panel EAMG group. From left to right is shown nAChR-staining (**red**), Homer staining (**green**) and a merged image of both staining. No significant changes between CTR and EAMG are observed in 3D analysis in neither *EDL* nor *GAS* in Homer endplate staining. Magnification bar = 10 µm.

A 3D-analysis of endplate-adjacent Homer was run for *EDL* and *GAS* comparably to *SOL*. Results showed that the mean quantity of Homer immune-signal pixels did not differ much in EAMG samples of both *GAS* and *EDL* compared to CTR (*GAS*: +8.97% MG vs. CTR; *EDL*: −0.58% MG vs. CTR) (Figure 5) in contrast to *SOL*.

To further separate from *SOL*, clinical subgroups of *GAS* and *EDL* did not show much difference in between them, even in clinically severely affected mice.

**Figure 6:**
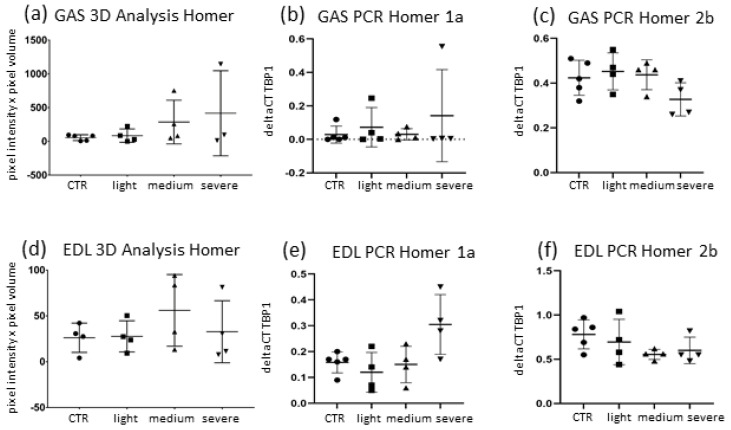
Homer protein expression in *EDL* and *GAS* muscle. (**a**) 3D analysis of *GAS* endplate Homer intensity and volume. No significant change of Homer is measured in *GAS* (+8.97%, *p* ≤ 0.27) between CTR and EAMG. (**b**,**c**) qPCR analysis of *GAS* showed no significant changes in mRNA Homer 1a expression in GAS (307%, *p* ≤ 0.905). (**d**) 3D analysis of *EDL* endplate Homer intensity and volume. No signs of change in Homer quantity in *EDL* endplates (0.58%, *p* ≤ 0.77). (**e**,**f**) qPCR analysis of *EDL* showed a trend towards an increase of short isoform Homer 1a mRNA (+87.5%, *p* ≤ 0.056) in severely affected mice, no change could be shown in other isoforms. Data are express as means ± SD.

qPCR data analysis of both fast-fibre dominant muscles revealed no significant changes of mRNA expression in either isoform, although trends of Homer 1a mRNA-induction were observed in both muscles (Figure 6b,c,e,f).

qPCR data analysis of *GAS* muscle revealed a trend towards an increase in short Homer 1a in severely affected animals compared to CTR animals and no considerable changes in the other Homer isoforms (Figure 6b,c). In *EDL* muscle, a trend in Homer mRNA increase could be seen in severely affected EAMG mice (Group IV) regarding the short dominant negative isoforms (Homer 1a: +87.5% compared to CTR, *p* ≤ 0.056) (Figure 6e), whilst long Homer isoform displayed constant or even reduced expression of mRNA without significance (Figure 6f). In addition, no significance is found in both *GAS* and *EDL* qPCR analysis.

Thus, compared to *SOL*, Homer 1a expression changes in mRNA are apparently less prominent in both *GAS* and *EDL* muscle, showing mean differences mostly in clinically severely (Group IV) affected mice. Similar changes in *SOL* were present already in medium affected mice (Group III).

Taken together, Homer mRNA expression seems to shift at an earlier stage of clinical manifestation in *SOL* than in the other two muscles.

**Figure 7:**
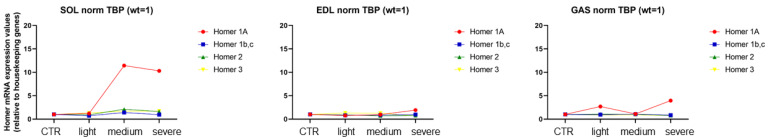
Overview qPCR analysis of homer gene isoforms in *SOL*, *EDL,* and *GAS* muscles of CTR and low, medium, severe clinical muscle weakness mice groups (*n* = 5, 4, 4, 4 mice per experimental group, respectively). An increase of Homer 1a in *SOL* is apparently associated with the increase of severe clinical muscle weakness. Data are normalised to TATA-box-binding protein (TBP1) and are expressed as means ± SEM over CTR. For calculation, comparisons were made by Mann–Whitney-U test, with *p* ≤ 0.05 being considered statistically significant.

In order to better follow the changes in the transcriptional regulation of Homer isoforms 1a, 1b-c, 2, and 3 in the *SOL* and *EDL* muscle of EAMG mice compared to CTR mice, the mRNA transcripts of the various Homer isoforms were shown in the same graph for comparison. As shown in Figure 7, a significant increase in transcription of Homer isoform 1a is clearly evident in the slow-twitch *SOL* muscle of the EAMG medium (Group III) and severe (Group IV), whereas only a trend was observed in the fast-twitch *GAS* and *EDL* muscle of the severe (Group IV) only group.

### 2.4. Homer Crosslinking Activity Differs in Muscles Depending on Dominant Fibre Type

To determine Homer protein concentration not only in proximity to individual NMJs, but also in the whole muscle, a Western blot (WB) analysis of *EDL* for mainly fast-type dominant muscle and *SOL* for mainly slow-type dominant muscle was done. Analysis was primarily done in native experimental conditions (in absence of reducing and denaturing reagents, keeping Homer protein–protein interaction intact) to distinguish between long isoform monomer, dimer, and multimer Homer concentration compared to CTR.

**Figure 8:**
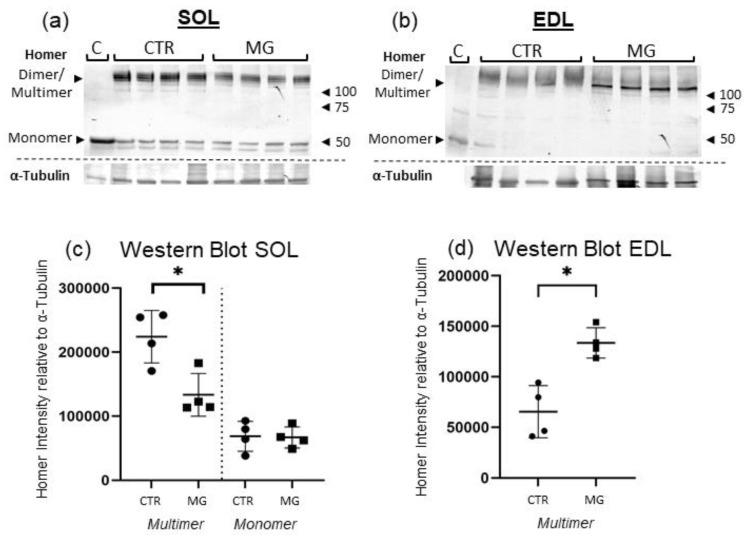
Homer Western Blot analysis of *SOL* and *EDL*. (**a**) WB total protein concentration of Homer in *SOL*. Band between 150 and 100 kDa represents Homer Dimers. Significant decrease of Dimer band intensity in *SOL* (−53.76%, *p* ≤ 0.0286) in native experimental conditions. (**b**) WB total protein concentration of Homer in *EDL*. In *EDL*, Homer 120 kDa band intensity is significantly increased (+146.25%, *p* ≤ 0.029) in EAMG compared to CTR. 42 kDa band, representing monomers, could not be detected in *EDL*, most likely due to relatively low expression of monomers in relation to Dimers. (**c**) WB of *SOL*. Dimers/Multimers between 150 and 100 kDa, Monomers (43–48 kDa) below 50 kDa. CTR samples 1–4 on the left, EAMG samples 1–4 on the right. α-Tubulin blot below was used for normalisation. (**d**) WB of *EDL*. All analysis was done in line with *SOL* Blot. Line C = Homer monomer positive control (murine cerebellum), *n* = 4 for CTR and EAMG. * indicates significant difference vs. CTR.

In WB, anti-panHomer antibodies identified different immunoreactive bands with a predicted Homer molecular weight in all muscle samples (Figure 8a,b). Bands were most prominent near 120 kDa and in some samples also seen near 43–48 kDa. Consequently, the 120 kDa band represents Homer dimer/multimers, while 43–48 kDa bands display Homer monomers (Figure 8a,b).

*SOL* Western blot analysis displayed a decrease of Homer dimer/multimer concentration (Figure 8a). The 120 kDa bands were significantly reduced, showing a decrease of 53.76% compared to CTR, whereas monomer band concentration remained unchanged in *SOL* muscle in native experimental conditions.

In contrast to *SOL*, densitometry analysis of the muscle protein bands in EDL indicated a significant increase of Homer 120 kDa band of 146.25% compared to CTR after normalisation (*p* ≤ 0.0286) (Figure 8b). Monomer muscle protein bands in native experimental conditions were only visible in one of the muscle blots (displaying a trend towards a Homer increase as well with a relative protein difference of +53.9% in MG muscle) and thus could not be reliably analysed in *EDL*.

WB of *EDL* samples in reducing experimental conditions are carried out to break apart Homer protein–protein interaction and ascertain the existence of monomers. Only the 45–48 kDa band could be detected (Figure S1). EAMG muscle sample Homer protein concentration is increased significantly by 416.49% compared to CTR.

Due to the limited amount of tissue from mice *SOL* muscle, no data are available in reducing experimental conditions.

Taken together, these results show that Homer Dimer is increased in fast-type dominant (*EDL*) and decreased in slow-type dominant muscle (*SOL*).

### 2.5. Muscle Fibre Cross-Sectional Area Is Decreased Only in EDL Muscle

To establish whether the differences reported so far among the different animal groups were influenced by muscle mass/atrophy, a morphometric analysis of fibre cross-sectional area (CSA) was carried out. Therefore, CSA of fibres in *GAS*, *EDL,* and *SOL* was determined. Dystrophin staining was used to define each fibre border whereas slow-type myosin heavy chain staining was used to identify Type-I muscle fibres. Negative slow-type myosin heavy chain staining was considered in Type-II muscle fibres.

**Figure 9:**
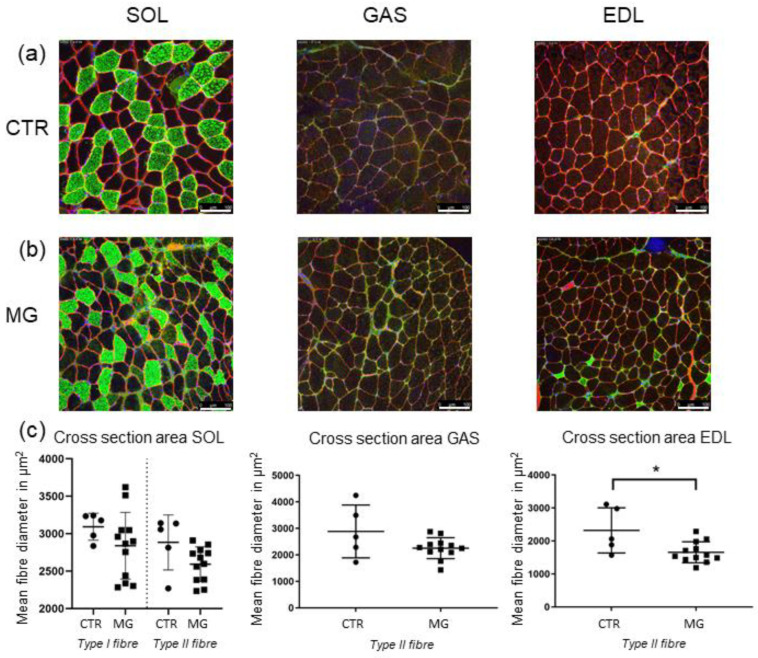
Muscle fibre cross-sectional area/morphometry analysis. (**a**,**b**) Staining of muscle sections of each *SOL*, *GAS,* and *EDL* with Dystrophin (red), Myosin slow (green) and DAPI (blue). The upper panel (**a**) representing CTR animals while lower panel (**b**) contains sections of EAMG-affected mice muscle. (**c**) Cross-section area statistical analysis: No significant reduction of muscle fibre diameter could be detected in both *SOL* and *GAS* muscle (SOL: *p* ≤ 0.08; *GAS*: *p* ≤ 0.23). In *EDL* (**c**), a significant reduction of mean fibre diameter could be detected in EAMG-animals compared to CTR group (*p* ≤ 0.037). In all three muscle groups absolute mean diameter is lower in MG than in CTR group. Type-I-fibres could only be statistically analysed in SOL muscle, in *GAS* and *EDL* samples slow-type fibres were not or only sparsely detectable. Thus, in *GAS* and *EDL* only Type-II-fibre diameter was analysed. In all analyses *n* = 5 for CTR, *n* = 12 for EAMG. * indicates significant difference *vs* CTR.

The results of dystrophin-staining (Figure 9a) showed a decrease of total mean fibre diameter in EAMG-affected animals in all three muscles: −9% in *SOL*; −15.4% in *GAS* and −22.2% in *EDL* (Figure 9c). A differentiation of fast-type *vs* slow-type fibre atrophy was only possible in *SOL* muscle, since slow-type fibres were rarely found in both *GAS* and *EDL*.

In *SOL* however, mean fibre diameter reduction was more severe in fast-type fibres (−13.7%) than in slow-type fibres (−9%). However, in *EDL,* slow-type fibres could only be detected in EAMG muscles but not in the CTR group, which might be suggestive for a possible fibre-switch in EDL (Figure 9c).

Statistical analysis of total muscle fibre diameter showed a significant atrophy only in *EDL* muscle (*p* ≤ 0.0365), while in both *SOL* and *GAS* no significant reduction was found (*GAS*: *p* ≤ 0.234; *SOL*: *p* ≤ 0.818)

A further analysis among the EAMG groups divided by clinical severity showed no significant atrophy differences in neither muscle.

### 2.6. Age Independent Localisation of Homer Expression and Postsynaptic Subcellular Localisation

To investigate whether differences in Homer endplate affection between old (ca. 78w) and young animals (approx. 30w) are present, endplate-adjacent Homer staining intensity was measured, and a comparison was made between old vs. young animals as well as between these two groups and CTR.

No significant differences could be detected between those two groups and CTR. When distinguishing between muscle types, *SOL* muscle presented no distinct difference in median pixel value observing young vs. old animals, whereas in *GAS* and *EDL* muscle a trend in median value with a lower Homer staining in older compared to younger animals was prominent (*GAS*: −20.6% young vs. old; *EDL* −26.8% young vs. old). However, since young animals in this group were only *n* = 3, this small sample number of young mice makes statistical significance between groups difficult to reach (Figure S2).

Interestingly, in these samples, Homer pixel intensity was significantly increased in both *EDL* and *GAS* muscle when comparing all EAMG samples to CTR, whereas *SOL* pixel intensity remained unaltered vs. CTR.

## 3. Discussion

The main objective of this study was to further investigate the Homer protein isoform expression pattern and crosslinking activity together with neuromuscular subcellular localisation in mouse hind limb muscles of an experimentally induced autoimmune disease of myasthenia gravis and to correlate to motor end plate integrity in both postural *(SOL)* and non-postural *(GAS, EDL)* muscles.

In previous studies, we showed that Homer protein family members are conserved in healthy mouse, rat, and human skeletal muscles, and are particularly localised at the postsynaptic microdomain, where they play a key role in coordinating the neuromuscular signal transduction machinery in a mechanism dependent on nerve and muscle activity [2,9]. We therefore set out to study the expression pattern of Homer genes and postsynaptic subcellular localisation in MG-affected skeletal muscles to find out whether they are linked to a proper expression and function of the nicotinic acetylcholine receptor (nAChR) in disease-affected peripheral synapsis.

We implemented this by using a destructive alteration of postsynaptic NMJ structures via EAMG mice and observing changes in Homer protein expression at whole muscle tissue and in situ at the NMJ postsynaptic subcellular localisation, thus correlating possible changes with the degradation of entire motor endplate structures.

As shown, the destruction of motor endplates in EAMG skeletal muscle is manifested mainly in *SOL* muscle rather than in *GAS* and *EDL*, and correlates with clinical severity. The main difference among the three hindlimb muscles being investigated (except for function) lies in their fibre type composition (*GAS* and *EDL*: mostly fast-type II, *SOL* mixed type I and II fibres). While *GAS* and *EDL* were mostly in line with each other, *SOL* results clearly differed from the other two muscles in terms of postsynaptic nAChR expression/cluster, Homer mRNA transcription, and protein expression/postsynaptic subcellular localisation.

It seems that rather than muscle function or location, nAChR regulation is dependent on muscle fibre-type composition, manifesting itself in both the affection of end plates as well as differences in postsynaptic Homer concentration, isoform quantity, and mRNA gene expression.

It is known that MG manifests itself with a stronger atrophic and clinical effect in fast-twitch type II fibres, although evidence available in the literature showed that it also affects slow-twitch type-I fibres [19]. Consistently, with this mouse model, *SOL* muscle was more severely affected than *GAS* and *EDL* fast-twitch muscles.

We also found that in the mixed-type *SOL* muscle, there was a decrease in Homer long isoform protein expression and crosslinking activity (decrease dimer/multimer) together with a decrease in Homer and postsynaptic nAChR, further accompanied by an increase in Homer 1a and Homer 2a/b gene transcription. In contrast, fast-type muscles such as *EDL* and *GAS* displayed an increase in Homer protein expression and crosslinking activity (increase in dimer/multimer) with a trend towards an increase in Homer 1a transcription and no change at the level of Homer 2a/b and nAChR.

Thus, substantial differences are present in terms of Homer crosslinking activity between the two types of EAMG muscles. The observed decrease in *SOL* is also supported by the increase transcription of Homer 1a the dominant negative isoform and a reduced expression of nAChR in motor endplates.

In previous studies, by using several experimental models of muscle disuse atrophy such as denervation, hindlimb unloading, and/or bedrest, it was shown that *SOL* muscle seemed to be more vulnerable to alterations in activity and motor endplate affection than *GAS* and *EDL.*

Although many factors, such as differences in cellular metabolism or contractile properties, could potentially be responsible for the different reaction between type-I and type-II fibres, we cannot fully exclude the hypothesis that the divergent effect of pathological EAMG mechanisms could directly affect Homer expression and regulation among muscle types through differences in impaired signalling mechanisms. Alternatively, compensatory mechanisms of synaptic transmission between fast- and slow-twitching muscle fibres might be present.

Different compensatory mechanisms seen in Myasthenia Gravis that are counteracting the impaired transmission mainly consist of a higher synthesis of nAChRs [20] as well as a higher number of ACh-quanta released from the presynaptic membrane following a single nerve impulse [21], counteracting the overall loss of nAChRs. This leads to a higher activity in single nAChRs.

Homer is known to play a key role in long-term potentiation and depression through modulation of mGluRs, especially mGluR1 and mGluR5 [22], and influencing central synaptic plasticity through those players [23].

Homer molecular structure showed a conserved Ena/VASP Homology 1 (EHV1) at the amino-terminal region, which is prominent in all Homer isoforms, and which selects proline-rich (PPXXF) amino acid sequence present in a multitude of postsynaptic protein interacting partners [24]. Proteins containing these sequences include metabotropic Glutamate receptors Group 1 (mGluR1), IP3-receptors (IP3R), transient receptor potential canonical (TRPC) receptors, and proteins involved in calcium signalling, such as the L-Type Ca^2+^ channels, the intracellular calcium release channel Ryanodine receptors (RyRs) and the nuclear factor of activated T cells c (NFATc), in addition to Phospholipase C-β and Shank [22,24,25,26,27].

The alteration of Homer in the EAMG affected motor endplates could therefore be the result of a compensatory effect caused by the altered force transmission, or a direct consequence of the change in activity in the postsynaptic nAChRs since Homer expression is also known to be muscle activity and thus nerve activity-dependent [2].

As seen in Western blots experiment results, Homer dimer quantity differed in *SOL* compared to *EDL*, showing no increase and even a trend of a decrease in overall protein in the whole muscle. In other muscle disuse models such as denervation, bed rest and hindlimb unloading, Homer 2a/b was shown to decrease especially in slow-twitch muscles as *SOL* as an early event of muscle disuse atrophy [10].

This would imply further evidence for the functional link between the pathogenic mechanisms of MG at NMJs and Homer protein.

Most striking in our qPCR analysis was the Homer isoform switch, which could be a result of neural damage. We saw an increase in short Homer 1a gene expression, which was especially prominent in *SOL* muscle. It is noteworthy to point out that Homer 1a, the dominant negative isoform, plays a key role in synaptic plasticity due to it uncoupling long isoform Homer from one another and its binding partners [16], being described as a fine-tuning mechanism in synaptic strength adaptation. Furthermore, Homer 1a can activate mGluR1 glutamate-independently, leading to a downregulation of synaptic strength [28,29]. Its expression leads to a remodelling of the PSD via an uncoupling of mGluRs from the scaffolding complex, resulting in a NMDA-current inhibition, whereas long isoform Homer creates the exact opposite effect [15]. NMDA-current inhibition of Homer 1a is a known mechanism to protect synapsis against neuronal injuries due to over excitation [30]. NMDA-induced Ca^2+^ influx and possible overload can be prevented via this switch in isoforms and has been demonstrated in traumatic brain injuries and ischemia [30]. Although mostly short-term based, a similar effect as a reaction to over stimulation of single receptors could play a key role also in EAMG pathology.

Unfortunately, our current knowledge about the molecular mechanisms involved in postsynaptic regulation of Homer is still incomplete. In skeletal muscle both mGLuR1 and NMDA are not expressed, which means that other molecular mechanisms and/or interacting partners must be in play. Therefore, further studies are necessary to investigate this part.

Another important effect of Homer 1a uncoupling is linked to reduced reactive oxygen species (ROS)-generation in peripheral synapses as well as the inhibition of downstream signalling [30]. It is highly implied that oxidative stress plays a role in pathogenesis of MG [31] and it is known that increased ROS can lead to nAChR-inactivation [32]. Thus, ROS-inhibition of Homer 1a could be a further protective mechanism that remains to be determined by further studies.

Interestingly, the EAMG mice subgroup showing the highest increase in Homer 1a mRNA transcript also showed significant reduction in nAChR-density in motor endplates. This group consisted of mice that exhibited the greatest clinical symptoms when examined prior to sample acquisition (group IV), suggesting that clinical severity is closely related to motor endplate disruption in these EAMG mice as expected. This also suggest Homer 1a overexpression is linked to both disease severity and extent of postsynaptic damage of NMJs.

Compared to data from WB whole muscle protein analysis, it, at first glance, appears that the results are not in line with one another. For example, the qPCR data showed an increase in the short Homer 1a isoform in *SOL* whereas in the *EDL* WB, the 120kDa dimer band was more prominent. This disparity is most likely caused by the reduction of Homer multimer protein complexes through the dominant negative short isoform, destroying the large networks and then splitting them into smaller dimer proteins, increasing the number of 120 kDa Homers. In fact, we see a decrease in the 120 kDa band in *SOL*, probably resulting from the previously described atrophy process [10]. In *SOL*, qPCR data showed an increase in Homer 2a/b mRNA specific for this muscle, perhaps due to a compensatory response for the loss of this protein isoform.

Most patients suffering from MG display antibodies (ABs) that are mainly against nAChRs, and are clustered at the postsynaptic folds of NMJs [33]. Other less frequent forms of MG include autoantibodies against muscle specific kinase (MuSK) and/or low-density lipoprotein receptor–related protein 4 (LRP4) [34,35]. Antibodies are usually of IgG1 or IgG3 subclasses and induce complement activation. This causes complement-mediated damage via membrane attack complexes (MAC), which leads to disarray of postsynaptic structures [36]. Moreover, ABs cross-link receptors at the postsynaptic membrane accelerating internalisation and turnover rate of nAChRs [33]. Reduction of post-synaptic nAChRs impairs synaptic transmission and causes a decrease in end plate potential (EPP). EPP decreases progressively at repetitive muscle contractions and ultimately falls below the threshold to trigger a propagated action potential [37].

Notably, inducible nitric-oxide synthase (iNOS, NOS2) and other MG-affected binding partners could possibly also have an influence on postsynaptic Homer expression in neuromuscular disease.

Muscle fibre-type composition seems to influence both MG pathology severity as well as Homer behaviour in affected muscles. A study by Krolick et al. [38] showed similar findings when investigating the role of iNOS and NO-production in EAMG animals, seeing a higher iNOS activity in slow-type fibres dominated *SOL* muscle coupled with a lower disease severity compared to *EDL,* as well as an increase in EAMG susceptibility when blocking iNOS. iNOS is a calcium-dependent enzyme [39], and an elevation of intracellular Ca^2+^ via cellular internal storage leads to an increased activity of iNOS via NO production, which is linked to mGluR plasticity and NMJ transmission modulation [40]. Subsarcolemmal iNOS/Caveolin-3 accumulations were also found mostly in type-1 fibres in human *Vastus Lateralis* (*VL*) and *SOL* muscle following extended disuse in bedrest [41]. iNOS activity is affected by calcium storage interaction via Homer scaffolds [1]. Since both metabolism and composition differ between fast and slow muscle fibres, a multitude of factors may explain the disparity of Homer expression pattern in these muscles, however, suggesting yet another unknown link between Homer and iNOS in healthy and diseased skeletal muscle that needs to be further investigated.

Finally, in about 54% of seronegative Myasthenia Gravis patients, anti-RyR1 (ryanodine receptor type 1)-antibodies can be detected [42]. Additionally, present in seropositive patients, these antibodies, as well as TRCP and Titin-Abs, are linked to a severe clinical disease manifestation [43,44]. Both RyR1 and TRCP are direct binding partners of Homer protein and play a role in the Ca2+ homeostasis of the post-synaptic NMJ [25,27]. Antibodies against these binding partners could potentially reduce a compensatory effect of Homer and/or imbalance Ca2+ regulation and in turn aggravate clinical symptoms via such putative pathological mechanism.

### Outlook and Limitations of the Study

One question that remains unanswered is whether Homer alteration could be the result of adaptation processes to altered transmission or part of the pathologic progress itself. Experiments with Homer 1 knockout in mice caused myopathy, most likely caused by a dysregulation of TRP channel activity leading to a disrupted Ca2+ homeostasis [17]. Since Homer seems to play a role in muscle strength maintenance, a lack of Homer would presumably aggravate muscle atrophy in MG affected patients. Should this prediction be confirmed in future experiments, it would make a compensatory effect of Homer much more likely rather than an enhancement of the MG pathological process.

All results in this study were gathered in the EAMG mice experimental model. EAMG shows very similar features in pathology but differs from normal *Myasthenia gravis* in ultrastructure [45]. Since MG is a chronic disorder, studies investigating different time points in disease progression could provide more information about the time course of observed Homer and AChR changes in the disease. EAMG induced mice also seemed to be more affected in type I-dominant fibres muscle from *SOL* than in type-II dominant muscles (GAS, EDL), which are usually primarily affected in MG patients. Further studies with human MG patients are mandatory to confirm if the present findings can be at least partly transferred from EAMG mice to real clinical pathology in terms of a further disease mechanism.

We are well aware that EAMG induction is not always successful, resulting in approximately 10–20% of non-responding mice without antibody-production [11]. This, coupled with individual vulnerability to disease severity [46], can blur possible effects in experiments with small sample or animal numbers like in the present observational/proof-of-concept-study. Further studies with higher sample/animal numbers are certainly required before considering studies in a clinical setting.

## 4. Materials and Methods

### 4.1. Animals

Adult C57BL/6J mice were use in all experiments, treated at the laboratory of the Department of Experimental Neurology Charité. All animal experiments were conduct in accordance with the European directive on the protection of animals used for scientific purposes and approved by the Charité ethics commission [47].

Induction of EAMG in mice was achieved by immunising mice with torpedo nAChRs (tAChR) antigens according to a previous established experimental protocol [48]. Mice were anaesthetised first with ketamine/xylazine or isoflurane and immunised subcutaneously with 40 μg of torpedo-purified acetylcholine receptor (tAChR) in the presence of a complete Freund’s adjuvant [47]. Two identical booster immunisations were performed four (t1 = 13 w) and eight (t2 = 17 w) weeks later. Pain medication (buprenorphine 0.05 mg/kg i.p. every 8 h or metamizole 200 mg/kg/d p.o.) were given for three days after each immunisation. An age-matched cohort of mice was used as a reference control group (CTR).

Disease scoring was carried out as previously described [47] according to standard methods used in the EAMG model [11] on a weekly basis. In short, apart from measures of general well-being (including body weight, body temperature, fur appearance, posture, and agility) muscle strength and fatigability were assessed by suspending the mice by the tail and allowing them to grab the bars of the cage grid. The gripping strength of their forelimbs was judged by an experienced investigator. Subsequently, the mice (still suspended by the tail) were moved across the top of the cage for 1 min, during which they were allowed to repeatedly grip the bars of the cage grid to induce forelimb exhaustion. Afterwards, gripping strength, posture and agility were re-assessed. Disease severity was evaluated on a five-point scale, where 0 = normal grip strength/no obvious abnormalities, 1 = no apparent muscle weakness before, but reduced strength after exercise; 2 = a noticeably decreased muscle strength already before exercise, which is further reduced after exercise with pronounced differences in posture and agility (hunchback); 3 = severe muscle weakness already before exercise; and 4 = paralysis.

Mice were euthanised at 20 and at 68 weeks after first immunisation to study both acute and chronic immunisation effects.

### 4.2. Muscle Samples Were Acquired in Two Different Batches

The first batch/group (*n* = 8) containing mice with low to medium clinical symptoms and two different age groups (young animals t = 30 weeks ± 1 week; old animals t = 78 ± 3 weeks).

The second batch/group (*n* = 12) consisting of mice with the same age (t = 28 weeks) but varying severity of symptoms, ranging from no (clinical score 0), medium to severe clinical muscle weakness. Each batch was compared to an age-matched control group (CTR) (batch 1: *n* = 4; batch 2: *n* = 5). CTR mice were treated in the same pattern as the EAMG mice with immunisations lacking the tAChR-Antigen. In total, EAMG group consisted of *n* = 20 mice, and CTR group were *n* = 9 mice.

### 4.3. Muscle Tissue Dissection and Preparation

Mice were euthanised via cervical dislocation, mice *soleus* (*SOL*), *gastrocnemius* (*GAS*), and *extensor digitorum longus* (*EDL*) muscles samples were taken, surgically dissected, from both left and right hindlimbs of each animal.

Muscles were snap-frozen in liquid nitrogen (N_2_). Right leg muscles that were used for immunohistochemistry (IHC) experiments were embedded in Tissue-Tek O.C.T. compound (www.laborversand.de; accessed on 30 November 2022) and snap-frozen in liquid nitrogen. Left leg muscles that were dedicated for mRNA extraction were snap-frozen and stored without O.C.T. compound. Both right and left leg muscles were stored at −80 °C for further processing.

### 4.4. Immunohistochemistry and Morphometry Analysis

Cross-oriented muscle cryosections with a thickness of 8µm were cut with a Leica CM 1860 cryostat (Leica Microsystems, Bensheim, Germany; www.leicabiosystems.com; uk.vwr.com accessed on 30 November 2022), mounted on a microscopy slide (Superfrost plus, VWR International, Radnor, PA, USA), and fixed with acetone-ethanol 1:1 *v/v* for 5 min at −20 °C. The samples were then washed three times with TBS (250 mM TRIS; 1.5 M NaCl; pH of 7.4.) 1:10 for 5 min, blocked with TBS 4% normal goat serum and 1% BSA for 1h and incubated with primary antibody (AB) diluted in TBS with 0.1% Triton X-100 for 1h at room temperature (RT).

For neutralising endogen primary monoclonal mouse antibodies, samples were additionally blocked with MOM-Block (Vector Laboratories, www.Biozol.de, accessed on 30 November 2022) for 1.5 h before applying the primary AB to reduce mouse-on-mouse AB interaction.

Muscle cryosections were then incubated overnight with primary antibodies. Slides were then washed three times with TBS for 5 min and incubated with fluorochromes conjugated secondary antibodies for 1 h at RT. Slides were washed four times with TBS for 5 min and mounted with DAPI mounting medium (D9542, www.sigmaaldrich.com, accessed on 30 November 2022).

If a second or third antibody was applied, the procedure was repeated as described for primary and secondary antibodies. After applying all antibodies, the samples were washed four times for 5 min with TBS, mounted as above described and stored overnight at 4 °C before laser confocal microscope analysis.

For the laser confocal analysis, a Leica SP-8 microscope was used (https://www.leica-microsystems.com, accessed on 30 November 2022). Digital images acquisition was done with the 20x and 40x objective, lasers used were 488, 555, and 405.

The muscle fibre diameters were analysed via SMASH (semi-automatic muscle analysis using segmentation of histology) by METLAB (Version 1.0). (https://www.mathworks.com/matlabcentral/fileexchange/59241-smashtoolbox-release, accessed on 30 November 2022). Fluorescence intensity and 3D-volume analysis were measured with the LAS X Leica Software (www.leica-microsystems.com, accessed on 30 November 2022).

3D imaging was done with LAS X 3D Analysis, endplates were digitally reconstructed via z-stack imaging, a threshold was applied to filter out background staining, volume, and mean intensity was measured automatically by the software and later multiplied to approximately quantify molecule density at endplates.

Primary Antibodies used were: goat anti-mouse Dystrophin (sc-73592 Santa Cruz Biotechnology, Dallas, TX, USA; www.scbt.com, accessed on 30 November 2022); goat anti-mouse _fast_Myosin heavy chain (My 32, Sigma M 4276, St Lous, MO, USA; www.sigmaaldrich.com/, accessed on 30 November 2022); goat anti-mouse _slow_Myosin heavy chain (Sigma M 8421; www.sigmaaldrich.com/, accessed on 30 November 2022); rabbit anti-pan Homer (Salanova’s lab, [8]). Primary antibodies were visualised by: goat anti-mouse Alexa Fluor^®^ 488 IgG (A11029, Invitrogen, Waltham, MA, USA, www.thermofisher.com, accessed on 30 November 2022); goat anti-mouse Alexa Fluor^®^ 555 IgG (Invitrogen A21424; www.thermofisher.com, accessed on 30 November 2022); goat anti-rabbit Alexa Fluor^®^ 488 IgG (Invitrogen, Carlsbad, CA, USA A-11008, www.thermofisher.com, accessed on 30 November 2022). Other reagents: α-Bungarotoxin 488-conjugated (B13422; www.thermofisher.com, accessed on 30 November 2022); α-Bungarotoxin 555-conjugated (B35451; Invitrogen, www.thermofisher.com, accessed on 30 November 2022).

### 4.5. Biochemical Protein Expression Analysis

To analyse Homer protein concentration, muscles were homogenised in a glass-dounce homogeniser in presence of RIPA-buffer (50 mM TRIS HCl with pH 7.4; 150 mM NaCl, 1% Nonidet P-40; 0.5% sodium deoxycholate; 0.1% SDS; 2 mM MgCl_2_) plus protease inhibitors in a ratio of 10:1 and investigated by biochemical Western blot analysis. The dissolved muscles were then centrifuged at 4 °C, 15,000 rpm for 25 min. The supernatant fraction, representing the solubilised protein fraction, was separated from the pellet or insoluble fraction and further analysed for the protein concentration.

For protein concentration determination, muscle solutions were diluted 1:5 with RIPA-buffer and afterward mixed with a colour reactive reagent in a ratio 8:1 of reagent to muscle-RIPA. The samples were mixed for 1 h on a shacking platform and afterward optically quantified. Protein levels of each muscle were quantified three times. The average protein concentration was calculated, and the samples were proportionally diluted to reach their protein concentration of 1 mg/mL proteins in 200 µL, the serum was aliquoted with the calculated amount of RIPA-buffer and 50 µL sample buffer. For native experimental conditions, sample buffer without reducing agents was used, and for reducing experimental conditions, ß-Mercaptoethanol and DTT were used.

For the SDS-Page, a precast gradient Mini-PROTEAN TGX gel by BIO RAD was used, SDS diluted 1:5 with purified water was used as a buffer for the chambers. Each gel pocket was filled with 15 µg, the gel was run for 1 h on 0.02 A and another 1 h to 1.5 h on 0.04 A. For the membrane transfer, the gel was transferred to a nitrocellulose membrane (0.45 mm), the transfer chamber was filled with 10% Towbin-buffer, and a constant voltage of 110 V was applied for 90 min. The chamber was cooled down to 4 °C during transfer. After the transfer, the membranes were stored overnight in TBS at 4 °C. The membranes were then incubated for 55 min in Blotto-buffer (4% skim milk powder in TBST) and afterward washed two times with TBST for 5 min. The membranes were then incubated with the primary antibody (Homer rabbit pAb [8] 1:500; α-Tubulin mAb 1:600 (Sigma T6199; www.sigmaaldrich.com, accessed on 30 November 2022)), which was dissolved in Blotto for 1 h at RT. They were washed 3 times with TBST and incubated with the secondary antibody (swine anti-rabbit 1:500 (DAKO, D0360; www.agilent.com, accessed on 30 November 2022) and rabbit anti-mouse DAKO 1:500 (D0314; www.agilent.com, accessed on 30 November 2022) for 1 h at RT dissolved in Blotto. Before colouring, the membranes were washed once in TBST, then once in TBS, and finally once in TBST + MgCl_2_ for 5 min each. For colouring, each membrane was incubated in 3.5 mL NBT with 10 mL TBS on the vibrator until bands could be seen. Finally, the membranes were washed with water and dried between blotting paper before scanning. α-Tubulin was used as an internal loading control (Sigma, T6199; https://www.sigmaaldrich.com, accessed on 30 November 2022) 1:600 as a housekeeping gene. Homer protein was normalised over α-Tubulin expression. Scanning was done with an Azure 400 visible Fluorescent Western System by Biozym^®^ (https://www.biozym.com/azure/, accessed on 30 November 2022). Densitometry analysis of the immunoreactive bands was further validated via ImageJ software (https://github.com/imagej/ImageJ, accessed on 30 November 2022).

### 4.6. Gene Transcription Analysis by Quantitative Polymerase Chain Reaction (qPCR) -RNA Extraction and cDNA Preparation

Total RNA was isolated from *SOL*, *EDL*, and *GAS* muscles (wild type group *n* = 5, group A (low severity) *n* = 4, group B (intermediate severity) *n* = 4, group C (high severity) *n* = 4) from each of the three muscles taken from adult mice. Frozen tissue samples were ground to a fine powder under liquid nitrogen and total RNA was extracted using TRIzol^®^ method, following the manufacturer’s instructions and including glycogen co-precipitating step. Reverse transcription was performed on 1 µg of total RNA by using SuperScript^®^ VILO™ cDNA Synthesis (ThermoFisher Scientific, Waltham, MA, USA).

Specific primers for Homer1a, Homer1b/c, and Homer2a/b were already published [49]. Primers for Homer 3 were designed with the Primer3 software (Whitehead Institute for Biomedical Research, Cambridge, MA, USA; http://frodo.wi.mit.edu/, accessed on 30 November 2022), and their thermodynamic specificity was determined using BLAST sequence alignments [U.S. National Centre for Biotechnology Information (NCBI), Bethesda, MD, USA] and Vector NTI^®^ (Invitrogen) Software. Homer 3 primer sequences were as follows: forward primer 5′-CACGCACTTACCGTGTCCTA-3′; reverse primer 5′-GGGAGTGACAGTGCTGTTGA-3′.

Primers’ sequences for TATA-box-binding protein 1 (TBP1) and Peptidyl prolyl Isomerase A (PPIA) are described in Bortoloso et al. 2013 [10] and Gambara G. et al. 2017 [50], respectively.

Quantitative PCR was performed in triplicate in a CFX96 Thermal Cycler (Bio-Rad, Hercules, CA, USA) using SYBR Green chemistry as previously described [10]. A melt-curve analysis was performed at the end of each experiment to verify that a single product per primer pair was amplified. Normalisation was performed by ΔCT method using Cyclophilin A (PPIA) and TATA-box-binding protein (TBP1) as reference genes. Data are expressed as means ± SD.

### 4.7. Statistical Analysis

Statistical analysis of muscles comparing EAMG to CTR groups were performed by Man–Whitney U test. Results of analysis were considered statistically significant at a predefined level of *p* ≤ 0.05. All analyses were done with Graph-Pad^®^ PRISM software (www.graphpad.com, accessed on 30 November 2022).

## 5. Conclusions

The expression pattern of postsynaptic scaffold adaptor proteins of the Homer family is differentially regulated in slow- and fast-type muscles of EAMG-affected mice.

Discrepancies were present at both biochemical and transcription level between the two types of muscle, suggesting a tight Homer regulation at protein translocation level from cytosol to postsynaptic subcellular compartments rather than an increase in relative protein expression.

The decreased Homer signalling in *SOL* muscle postsynaptic endplates of EAMG mice might be the result of a skeletal muscle adaptation process due to the impaired acetylcholine receptor signalling. Nevertheless, our study results provide compelling evidence for the existence of a functional coupling between Homer and nAChR function and activity in a murine EAMG animal model.

These findings, if confirmed in human MG patients, highlight the yet underestimated role of the Homer protein family in muscle cell neurophysiology and neuromuscular disease.

## Data Availability

Not applicable.

## Supplementary Materials

The supporting information can be downloaded at: https://www.mdpi.com/article/10.3390/ijms232315052/s1.
