# Supplementary material for: Opposite Regulation of Homer Signal at the NMJ Postsynaptic Micro Domain between Slow- and Fast-Twitch Muscles in an Experimentally Induced Autoimmune Myasthenia Gravis (EAMG) Mouse Model
Source: Int J Mol Sci. 2022 Nov 30;23(23):15052. doi: 10.3390/ijms232315052 (PMC9738765; doi:10.3390/ijms232315052)
Supplement: Supplementary file 1 [file ijms-23-15052-s001.zip › ijms-1997772-supplementary.pdf]

Western Blot EDL reducing experimental conditions

(a)

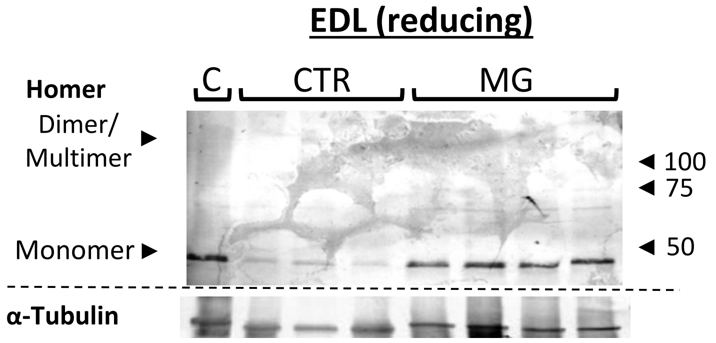

(b)

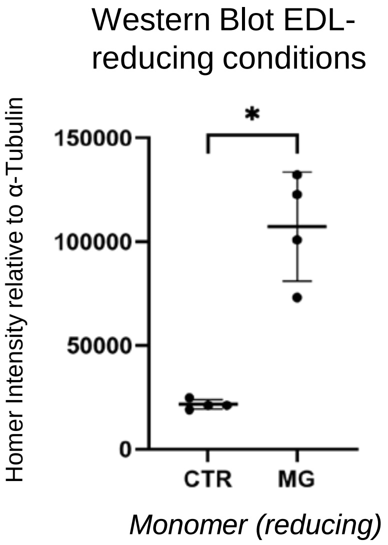

Supplement Figure S1:
Homer Western Blot analysis of *EDL* in *reducing* conditions.
(a): WB of *EDL*. Dimers/Multimers between 150 and 100kDa, Monomers (43-48kDa) below 50kDa. CTR samples 1-3 on the left, EAMG samples 1-4 on the right. α-Tubulin blot below was used for normalization. Line C= Homer monomer positive control (murine cerebellum).
(b): WB total protein concentration of Homer in *EDL*. In *EDL reducing*, Homer 42kDa band intensity is significantly increased (+416.49% %, $p<0.05$) in EAMG compared to CTR. 120kDa band, representing multimers, is not present in *EDL* due to reducing detergents splitting up protein-protein interaction. * = indicates significant difference *vs* CTR (ANOVA $p \leq 0.05$).

OLD *vs* YOUNG

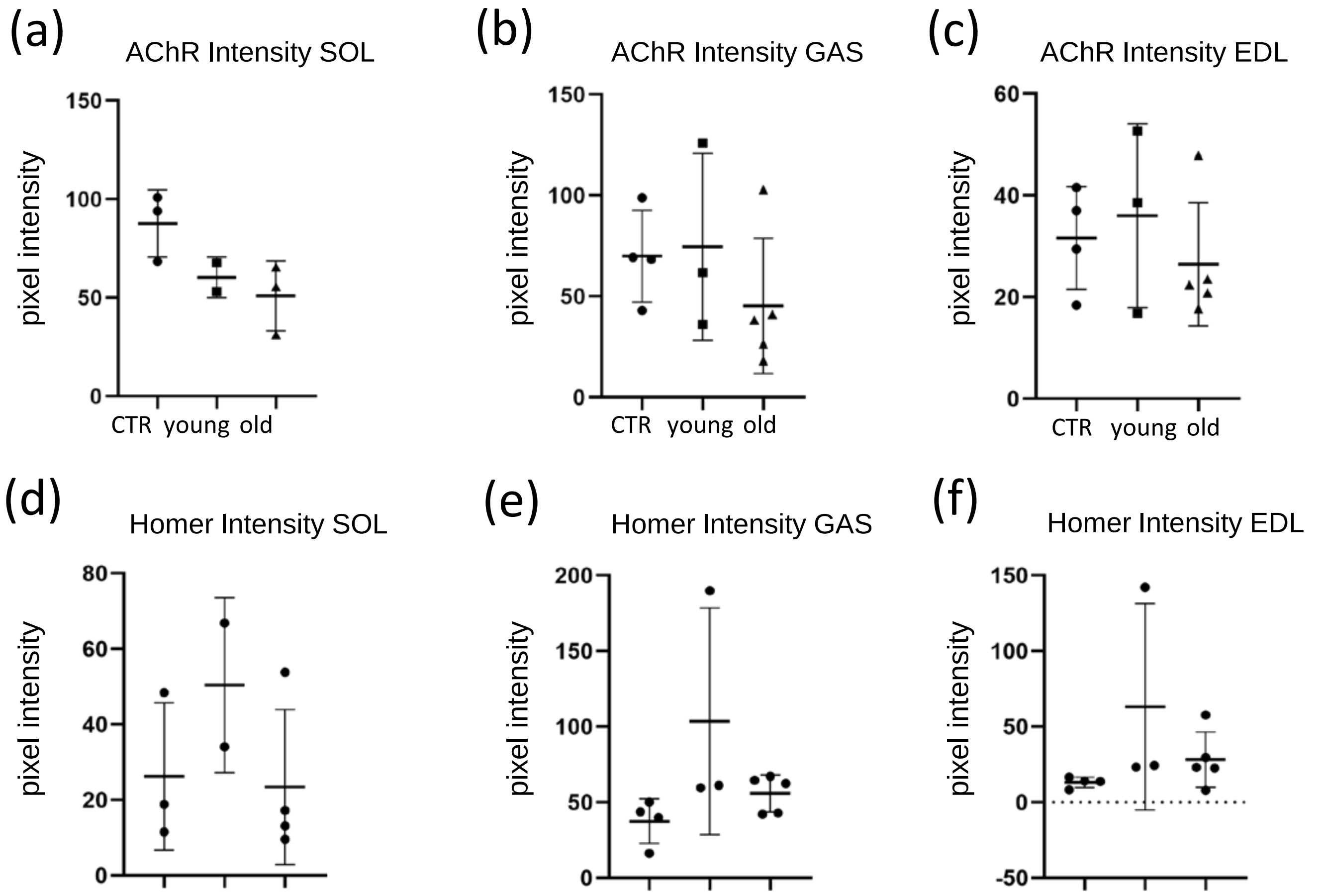

Supplement Figure S2:
nAChR degradation rate in endplates of hindlimb muscles EAMG *vs* CTR (a–c) and endplate-adjacent Homer protein (d–f) divided by age in young (28W) and old (70+w) mice. Results of confocal pixel intensity analysis, Alpha Bungarotoxin (αBTX) staining and Homer antibody staining. No significant results were seen in between age groups for both αBTX and Homer staining in neither of the three muscles.
Groups were n=4 for CTR, n=3 for young and n=5 for old mice.
Statistical differences between groups were determined by Mann-Whitney-U-Test. Significance *vs* CTR was considered at $p \leq 0.05$ (ANOVA).
